# Dietary Antioxidants May Support Cosmetic Treatment in Patients with Rosacea

**DOI:** 10.3390/antiox13030381

**Published:** 2024-03-21

**Authors:** Kinga Zujko-Kowalska, Joanna Masłowska, Małgorzata Knaś-Dawidziuk, Jadwiga Hamulka, Małgorzata Elżbieta Zujko

**Affiliations:** 1 Department of Cardiology and Internal Medicine with Cardiac Intensive Care Unit, Medical University of Białystok, M. Skłodowskiej-Curie 24a, 15-276 Białystok, Poland; kinga.zujko-kowalska@sd.umb.edu.pl; 2 Department of Cosmetology, Faculty of Health Sciences, University of Łomża, Akademicka 14, 18-400 Łomża, Poland; jmaslowska@al.edu.pl (J.M.); mknas@al.edu.pl (M.K.-D.); 3 Department of Human Nutrition, Institute of Human Nutrition Sciences, Warsaw University of Life Sciences (SGGW-WULS), 02-787 Warsaw, Poland; jadwiga_hamulka@sggw.edu.pl; 4 Department of Food Biotechnology, Faculty of Health Sciences, Medical University of Białystok, Szpitalna 37, 15-295 Białystok, Poland

**Keywords:** rosacea, quality of life, antioxidant treatment, lifestyle, dietary antioxidant quality index

## Abstract

Rosacea is a chronic skin disease that significantly reduces the quality of life (QoL) of patients. The aim of this study was to assess whether dietary antioxidants can support the effect of cosmetic treatment in improving the QoL of patients with rosacea. Basic data about participants (N = 160) were collected using a self-reported survey. QoL was assessed using the standardized Dermatology Life Quality Index (DLQI) questionnaire. An interventional cosmetic treatment was performed using cavitation peeling and sonophoresis with a commercial capillary repair serum. The study was registered as a clinical trial (NCT06271135). To assess antioxidant diet quality, for the first time, a new dietary antioxidant quality index (DAQI) was developed, which consisted of 12 items: dietary total antioxidant capacity, dietary polyphenols, phytosterols, lignans, vitamin C, vitamin E, β-carotene, zinc, iron, copper, manganese and selenium. It was found that the highest tertiles of DAQI diminished the odds about 40–45% of the moderate, large and extremely large effect of rosacea on QoL and about 8–11% of the occurrence of rosacea symptoms compared with the lowest tertile. This study showed that rosacea has a negative impact on QoL, but a comprehensive approach to treatment, including antioxidant cosmetic treatment and dietary antioxidants, can improve the QoL of patients with rosacea.

## 1. Introduction

Rosacea is a chronic inflammatory skin disease with a multifactorial and not fully understood etiology. Possible causes of the disease include genetic, immunological, infectious and environmental factors. Other causes include imbalance in the skin microbiome, gastrointestinal diseases and medications. Lifestyle, including poor diet, intense or low physical activity, stress, smoking, insufficient night sleep duration, excessive alcohol consumption and improper skin care, also play an important role in the development and treatment of rosacea. Its characteristic symptoms are recurrent flushing, erythema, telangiectasia, papules and pustules, localized mainly in the central part of the face and occurring with periods of remission and subsequent relapses [1,2]. There are four clinical subtypes of the disease: erythematotelangiectatic rosacea, papulopustular rosacea, phymatous rosacea and ocular rosacea [3]. Rosacea affects about 5% of the world’s population between 30 and 50 years with fair skin, living in the northwestern part of Europe with I or II skin phototype according to the Fitzpatrick scale [4]. Rosacea significantly reduces the quality of life (QoL), affecting the patient’s physical, mental and social well-being [5]. Therefore, a multi-aspect care strategy for a patient with rosacea must include pharmacological and cosmetic therapy as well as education regarding lifestyle changes [6]. Rosacea is difficult to treat and requires the cooperation of dermatologists, cosmetologists and dieticians. The cosmetologist is often the first person to notice not only dilated blood vessels, but also to diagnose the onset of the disease [7,8]. Among the cosmetic procedures in rosacea, the following can be distinguished: dermo cosmetics, several types of light and laser therapy, cavitation peeling, low-density micro-focused ultrasound, introduction of active substances (amino acids, antioxidants) using mesotherapy, micro needling, sonophoresis and ultrasound [9,10,11,12].

Appropriate eating habits also play an important role in the treatment of rosacea. Some foods may exacerbate the symptoms of the disease, such as alcohol, spicy and fried foods, chocolate, coffee, citrus fruits, refined sugar and milk. Others, such as vegetables, nuts, wholegrains, tea, and fish, may alleviate the symptoms of the disease [13].

In recent years, the role of oxidative stress in the pathogenesis of rosacea has been emphasized. The overproduction of reactive oxygen species in oxidative stress can cause cellular damage that leads to a variety of skin diseases. Natural exogenous antioxidants, such as polyphenols, antioxidant vitamins (C, E), carotenoids and minerals (zinc, iron, copper manganese and selenium) may support the action of endogenous antioxidants in mitigating the destructive effects of oxidative stress [14].

The results of many scientific studies confirm that rosacea has a negative, usually moderate impact on the quality of life, which significantly deteriorates as the severity of the disease increases, and in the presence of factors that worsen the symptoms of rosacea [15,16,17]. Therefore, the current therapeutic approach to treating rosacea is to combine different treatments, use appropriate skin care products, and avoid triggers that exacerbate symptoms. In addition, special attention is paid to lifestyle elements, including an appropriate diet rich in antioxidants. Some authors compared the effect of ivermectin 1% cream and metronidazole 0.75% cream in the treatment of severe papulopustular rosacea, showing better effectiveness of ivermectin 1% in improving the QoL [18]. Others showed that the use of a dermo cosmetic containing the sap of oat plantlets and mandarin extract alleviates the symptoms of rosacea and improves the QoL [19]. In our interventional study, we used a dermo cosmetic with high antioxidant properties. Additionally, in this study, for the first time, we analyzed the diet of patients with rosacea in terms of antioxidant content. For this purpose, a new dietary antioxidant quality index (DAQI) was developed and used, which included 12 elements: dietary total antioxidant capacity, dietary polyphenols, phytosterols, lignans, vitamin C, vitamin E, β-carotene, zinc, manganese, iron, copper, and selenium. There are no studies on the impact of dietary antioxidants on improving the quality of life of patients with rosacea. 

The aim of this study was to assess whether dietary antioxidants can support the effect of antioxidant cosmetic treatment in improving the QoL of patients with rosacea. 

## 2. Materials and Methods

### 2.1. Ethical Approval 

The study was conducted in accordance with the Helsinki Declaration and Good Clinical Practice, as well as approved by the Ethics Committee of the Łomża State University of Applied Sciences (Łomża (protocol code 517101, date of approval 5 May 2017)). Informed consent was given by all participants of the study. The study was registered as a clinical trial (clinical trial identifier: NCT06271135). 

### 2.2. Study Population and Data Collection 

The study population consisted of 160 patients (123 women and 37 men) with rosacea, who agreed to participate in this study. The inclusion criteria were as follows: diagnosis of rosacea according to the criteria of the National Rosacea Society Expert Committee (NRSEC) from 2019 [20], tolerance to the ingredients of the commercial capillary repair serum used in the cosmetic intervention, lack of pregnancy and lactation, lack of neuropsychiatric diseases and cancer, completion of a survey, signed informed consent. Exclusion criteria were as follows: patients with other facial skin diseases (e.g., acne vulgaris, psoriasis, eczema), intolerance to the ingredients of the capillary repair serum, patients with neuropsychiatric diseases, cancer, pregnancy, lactation, patients who did not sign informed consent to participate in the study and did not complete the survey. 

Patients were qualified for the study among people visiting the cosmetic studio in the city Choroszcz (Poland) in the period from June 2017 to November 2018, based on the diagnosis of a dermatologist and a cosmetologist. Study participants completed a survey questionnaire containing questions about sociodemographic status, anthropometric data, health behaviors, lifestyle, dietary habits and information about rosacea. The physical activity was assessed by a short version of the International Physical Activity Questionnaire (IPAQ) [21]. The assessment of QoL in rosacea was carried out using the Polish version of the standardized questionnaire DLQI (The Dermatology Life Quality Index). The DLQI is a 10-item questionnaire regarding patients’ perception over the previous week, with a total score of 0–30 points (0–1 point—no effect, 2–5 points—small effect, 6–10 points—moderate effect, 11–20 points—very large effect, 21–30 points—extremely large effect on the patient’s life) [22,23].

### 2.3. Dietary Antioxidant Quality Index (DAQI) 

Dietary habits were estimated using a 3-day nutritional interview. The energy value of the diet and the intake of antioxidant vitamins (C, E), β-carotene, and minerals (zinc, manganese, iron, copper) were assessed using Diet 6.0 computer program. The selenium content in the diet, dietary total antioxidant capacity (DTAC), dietary polyphenols (DP), dietary phytosterols (DPH) and dietary lignans (DL) were estimated using available databases [24,25,26,27,28,29,30].

A new DAQI (dietary antioxidant quality index) was developed, which consisted of 12 items. The final DAQI results ranged from 0 to 12 points and were classified as follows: low (1–4 points), moderate (5–8 points), and high (9–12 points) antioxidant quality of the diet.

The following cut-off values were adopted:

- DTAC, DP, DPH, DL, β-carotene/1000 kcal of diet ≥ mediane—1 point, <mediane—0 point,
- antioxidant vitamins: ≥90% RDA (recommended dietary allowances) for vitamin C or ≥90% AI (adequate intake) for vitamin E—1 point, <90% RDA or AI—0 point,
- antioxidant minerals: ≥90% RDA for zinc, iron, copper and selenium or ≥90% AI for manganese—1 point, <90% RDA or AI—0 point.

The RDA and AI of vitamins and minerals have been established based on the standards for the Polish population, which take into account the recommendations of experts from the European Food Safety Authority (EFSA), the World Health Organization (WHO), the Department of Health and Medicine Office for the United States and the results of the latest research [31].

### 2.4. Cosmetic Intervention with Antioxidant Commercial Capillary Repair Serum

Cosmetic procedures were performed in a group of patients after confirming the lack of contraindications to the procedure. The treatment was performed 3 times at 2-week intervals and consisted of cavitation peeling and sonophoresis with a commercial capillary repair serum. The F-808 Skin Scrubber device (Silverfox Beauty Salon Equipment Co., Ltd., Guangzhou, China) was used for the treatment. After a month, the procedure was repeated. The stages of a cosmetologist’s procedure during a cosmetic intervention were as follows: 1. make-up removal; 2. skin diagnostics, exclusion of contraindications, discussion of the procedure; 3. cavitation peeling–gentle skin cleansing using ultrasound; 4. applying a capillary repair serum (2 g); 5. performing sonophoresis treatment; 6. applying the cream (1 g) at the end; 7. discussion of indications for further skin care. 

A commercial capillary repair serum contained the following ingredients on its label:

aqua (water), glycerin, troxerutin, propylene glycol, aesculus hippocastanum (horse chestnut) seed extract, arnica montana flower extract, ascorbyl glucoside, hydrolyzed caesalpinia spinosa gum, caesalpinia spinosa gum, lactic acid, hydroxyethylcellulose, glyceryl polyacrylate, alcohol denat, potassium hydroxide, peg-40 hydrogenated castor oil, phenoxyethanol, potassium sorbate, sodium benzoate, methylparaben, propylparaben, mentha arvensis leaf oil, citrus limon (lemon) peel oil, cupressus sempervirens leaf oil, lavandula hybrida oil, cistus ladaniferus oil.

The antioxidant potential of the commercial serum was determined using the ABTS method—TAS kit (Cayman Chemical Company, Ann Arbor, MI, USA) and expressed as mg Trolox Equivalent (TE) per 30 g of cream. The assay relies on the ability of antioxidants in the sample to inhibit the oxidation of ABTS (2,2′-Azino-di-[3-ethylbenzthiazoline sulphonate]) to ABTS• by methmyoglobin. Absorbance was read at 405 nm on a BioTek Epoch spectrophotometer. Before the assay procedure, samples (*n* = 3) were prepared as follows: 1 g of cream sample was extracted using hot water extraction and sonication procedure [32].

It was shown that the antioxidant capacity of used commercial serum was 48.73 ± 5.81 mg TE/30 g of cream.

### 2.5. Statistical Analysis

Statistical analyses were performed using IBM SPSS Statistics v. 27.0 software (SPSS INC., Chicago, IL, USA). *p*-values of less than 0.05 were considered statistically significant. Continuous variables were presented as mean (M) and standard deviation (SD) and categorical variables as count (N) and percentage (%). Categorical variables were compared with the Pearson’s chi-squared test. Comparisons of continuous variables between groups were conducted using the Mann–Whitney–Wilcoxon or Kruscal–Wallis tests. Normality of continuous data distribution was verified with the Shapiro–Wilk test. Logistic regression models were used to assess the relationship between DLQI and DAQI, as well as the occurrence of rosacea symptoms (pustules and papules, flushing, persistent erythema, and telangiectasia) after the cosmetic procedure and DAQI. Three models were performed: 1. Model—crude data, 2. Model—data adjusted for age, sex and daily energy intake; 3. Model—data adjusted for variables in Model 2, BMI and smoking. Results were presented as ORs (odds ratios) and 95% CIs (confidence intervals) of the association between the tertiles (Q1–Q3) of DAQI and DLQI score or the occurrence of rosacea symptoms after the cosmetic procedure.

## 3. Results

The baseline characteristics of the study population are shown in Table 1. The mean age was 46.4 ± 6.4 years (range 32–72 years); 77% were women, and 63% were under 50 years of age. Most participants had higher and middle level of education (79%). A large percentage of people had normal body weight (59%), but 24% were overweight and 14% were obese. A large group of people were characterized by an unhealthy lifestyle: 59% smoked cigarettes, 23% drank alcohol once a week or more often, 63% slept less than 7 h or more than 9 h, and only 48% had moderate level of physical activity. Almost all respondents (89%) had skin phototype I or II according to the Fitzpatrick scale. Some subjects were characterized by the presence of comorbidities, mainly digestive system diseases, *Helicobacter pylori* and hormonal disorders. Over 61% of people have struggled with rosacea for 4 years or longer. The first symptoms of the disease appeared at the mean age of 42 years (range: 16–52 years). The current condition of the patients’ skin included mainly: flushing, persistent erythema, telangiectasia, pustules and papules. The respondents indicated that the disease lesions were intensified by the following factors: sun exposure, temperature changes, some cosmetics, alcohol, spicy foods, hot foods and drinks, stress and intense physical activity. Among the analyzed group, 68% of people confirmed the presence of rosacea in their family.

Dietary antioxidant quality index and individual antioxidants, such as polyphenols, phytosterols, lignans, vitamins C and E, β-carotene, minerals such as zinc, iron, copper, manganese and selenium, and dietary total antioxidant capacity were presented in Table 2. It was found that most participants consumed antioxidant vitamins and minerals with their diet in amounts inconsistent with recommended doses. Consumption below 90% of the RDA/AI has been shown for vitamin C and E (81% and 89% of patients, respectively), as well as for zinc, iron, copper, manganese and selenium (67%, 57%, 56%, 60% and 78% of patients, respectively). The mean DAQI was 7.2 ± 3.9 (range: 3–11), which indicates a moderate antioxidant quality of the diet.

Associations between sociodemographic and lifestyle characteristics and DLQI were presented in Table 3. Statistically significant differences in the DLQI score were observed for age, BMI, smoking, alcohol drinking, night sleep duration, and DAQI. Rosacea had a greater impact on the QoL in women and younger people compared to men and older people. Among the modifiable lifestyle factors, the better QoL of people with rosacea was influenced by normal BMI, not smoking, not drinking alcohol, good sleep hygiene and a diet rich in antioxidants.

**Figure 1:**
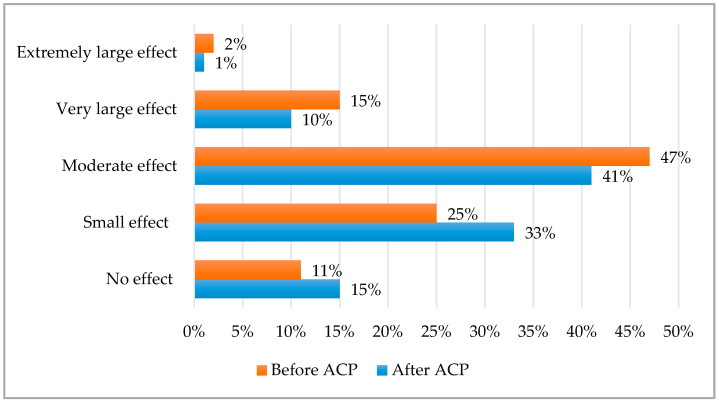
DLQI before and after ACP.

DLQI before and after the antioxidant cosmetic procedure (ACP) are shown in Figure 1.

It was found that the applied cosmetic procedure had a significant (*p* < 0.001) effect on the lower impact of dermatology disease on the QoL. Before ACP, DLQI was 9.2 ± 4.9, and after ACP, it was 7.6 ± 4.2. Before ACP, rosacea had a moderate, very large and extremely large effect in 47%, 15% and 2% patients, respectively. After ACP, a moderate, very large and extremely large effect was observed only in 41%, 10% and 1% patients, respectively. A small effect of rosacea on QoL was detected in 25% patients before and in 33% patients after ACP, whereas no effect of rosacea on QoL was observed in 11% people before and 15% people after ACP.

**Figure 2:**
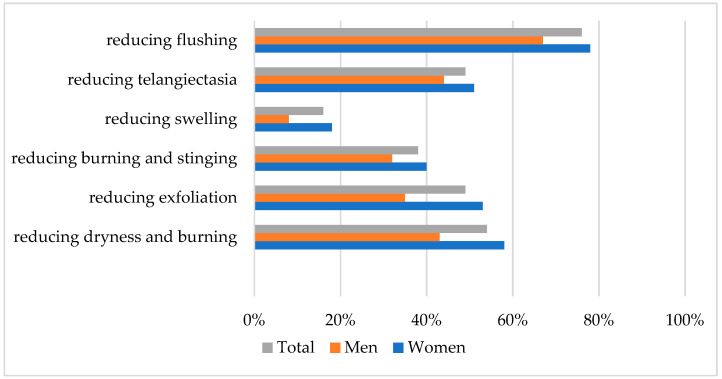
The most frequently observed positive changes after cosmetic treatment.

Positive effects after cosmetic treatment were noted by 80% of patients in subjective assessment. The most frequently observed positive changes by participants were presented in Figure 2.

Association between sociodemographic and lifestyle characteristics according to DAQI tertiles were presented in Table 4. It was found that, compared to the lower DAQI tertiles, a greater percentage of people in the higher DAQI tertiles were women, younger people, people with a lower BMI and non-smokers. No associations were found between the level of education, alcohol drinking, night sleep duration and physical activity.

The odds ratios for the impact of rosacea on QoL (DLQI) by tertiles of DAQI were evaluated using multiple logistic regression analysis (Table 5). Three models were tested: model 1—crude data; model 2—data adjusted for age, sex, daily energy intake, model 3—data adjusted for age, sex, daily energy intake, BMI and smoking. The first tertile (T1) in each model was adopted as a reference. It was found that in fully adjusted models, independently of confounding variables (age, sex, daily energy intake, BMI, smoking), the highest tertiles of DAQI diminished the odds about 45% and 40% (respectively) of the moderate (DLQI = 6–10), large and extremely large (DLQI > 10) effect of rosacea on QoL after cosmetic procedure compared with the lowest tertile of DAQI (OR = 0.55, 95% CI: 0.23–0.92 for moderate effect of rosacea on QoL and OR = 0.60, 95% CI: 0.22–0.97 for large and extremely large effect of rosacea on QoL). No association was found between the small effect of rosacea on QoL (DLQI < 6) and higher tertiles of DAQI.

The odds ratios for the occurrence of rosacea symptoms after cosmetic procedure by tertiles of DAQI were evaluated using multiple logistic regression analysis (Table 6). Three models were tested and the first tertile (T1) in each model was adopted as a reference. It was found that, in fully adjusted models, the highest tertiles of DAQI diminished the odds about 8%, 7%, 11%, 9% (respectively) of the occurrence of pustules and papules (OR = 0.92, 95% CI: 0.75–0.98), flushing (OR = 0.93, 95% CI: 0.72–0.97), persistent erythema (OR = 0.89, 95% CI: 0.75–0.96), and telangiectasia (OR = 0.91, 95% CI: 0.72–0.97) compared with the lowest tertiles.

## 4. Discussion

Rosacea is a serious social problem that significantly reduces the QoL; therefore, assessing the QoL of patients with rosacea and identifying factors that improve the QoL is an important element of clinical practice [33]. A comprehensive, interdisciplinary approach to the disease, including pharmacological treatment, cosmetic procedures and lifestyle education, can guarantee satisfactory results [34]. The pathophysiopathology of rosacea is multifactorial, and the most important factors include a positive family history, genetic susceptibility, disturbance of immunological response, neurogenic inflammation and dysregulation of the neurovascular system, and microorganisms [35]. In this study, 2/3 of patients confirmed the presence of rosacea in the family, which is consistent with other studies [36,37]. In addition to rosacea, patients had comorbidities, such as digestive system diseases, *Helicobacter pylori*, demodicosis, and hormonal disorders. Previous research has shown an association between gut microbiota and rosacea [38].

This study showed that rosacea has a negative impact on QoL, but a comprehensive approach to treatment, including antioxidant cosmetic treatment and a healthy lifestyle, especially dietary antioxidants, can improve the QoL of patients with rosacea.

There was no significant impact of the level of education and physical activity on the QoL of patients. However, it was shown that younger people and women suffered the effects of the skin disease to a greater extent, which resulted in their lower QoL compared to men and older people. Other authors also confirmed that chronic skin diseases pose a significant psychological burden at a younger age, causing a higher incidence of anxiety and depression [39]. For women and younger people, the condition of the skin is very important because it makes it easier to establish social relationships, while older people and men accept life limitations more easily [40]. A study conducted in China showed that young patients with rosacea, especially those with occupational requirements related to appearance, were more likely to have reductions in QoL [41]. Another Chinese cross-sectional study of 8700 patients with 16 skin diseases confirmed that the female gender, young age and alcohol consumption influence the lower QoL associated with dermatological diseases. However, the authors did not demonstrate such a relationship in the case of education, employment and BMI [42].

In our study, among the modifiable lifestyle elements, the most important were as follows: proper body weight, not smoking, not drinking alcohol, proper sleep hygiene and a high intake of dietary antioxidants, which significantly improved the QoL of patients with rosacea. Some authors indicate that positive lifestyle changes, mainly related to diet, sleep hygiene, moderate physical activity and social relationships, have a significant impact not only on health in general, but also on skin health [43].

There is still a lack of research assessing the relationship between rosacea and nutritional factors. The most commonly reported triggers for rosacea include alcohol, hot drinks, spicy foods, fatty foods, foods containing cinnamaldehyde, and foods high in histamine. However, some nutrients, such as omega-3 acid and zinc, are considered beneficial in reducing rosacea [44,45]. In this study, patients reported the following factors intensifying the symptoms of rosacea: sun exposure, temperature changes, some cosmetics, alcohol, spicy and hot foods and drinks, stress, intense physical activity.

The impact of cosmetic treatments on improving the QoL of patients with rosacea is rarely studied [18,19,46]. This study involved a cosmetic treatment consisting of cavitation peeling and sonophoresis with a commercial capillary repair serum with high antioxidant capacity. The cream used in the cosmetic intervention contained many natural ingredients, such as horse chestnut seed extract, arnica montana flower extract, mentha arvensis leaf oil, lemon peel oil, cupressus sempervirens leaf oil, lavandula hybrida oil, cistus ladaniferus oil, as well as troxerutin and ascorbyl glucoside. The ingredients of the preparation have a beneficial effect on vascular skin with rosacea because they are a rich source of antioxidants, such as vitamin C, E, carotenoids, and polyphenols. Moreover, arnica montana strengthens the walls of capillaries, horse chestnut stimulates blood circulation and elasticizes and strengthens blood vessels, vitamin C has a sealing and strengthening effect on blood vessels, and troxerutin reduces the permeability of blood vessels. Some authors have confirmed the effectiveness of topical antioxidants in improving skin condition [47,48,49]. This study showed that an antioxidant cosmetic treatment significantly improved the QoL of patients with rosacea.

This study indicates, for the first time, the important role of dietary antioxidants, along with topical antioxidants, in improving the QoL of patients with rosacea. Oxidative stress is involved in the pathophysiology of rosacea because it is associated with inflammation, vascular changes, and oxidative tissue damage [50]. The cutaneous antioxidant system consists of enzymatic (glutathione peroxidase, catalase and superoxide dismutase) and non-enzymatic low molecular weight antioxidants (estradiol, melatonin, vitamin E and C) [51]. Exogenous dietary antioxidants may support the action of endogenous antioxidants in mitigating the effects of oxidative stress [52]. Several antioxidant agents, such as vitamin C, E, carotenoids and polyphenols, have demonstrated efficacy in neutralizing ROS to prevent oxidative damage associated with inducing or irritating various dermatoses. Antioxidant supplementation has shown efficacy in treating cancer and non-cancer dermatoses, including rosacea, psoriasis, atopic dermatitis, and acne vulgaris [53]. Some authors have shown that after oral supplementation with vitamins C and E, they accumulate in the skin layers. However, the distribution and accumulation of orally ingested antioxidants in the skin may vary depending on the type of antioxidant and individual factors [54,55,56]. It is important to note that the use of oral supplements or topical antioxidants does not replace a diet rich in antioxidants. In this study, to assess the antioxidant quality of the diet, a new dietary antioxidant quality index (DAQI) was developed. DAQI consists of 12 elements: dietary total antioxidant capacity, dietary polyphenols, phytosterols, lignans, vitamin C and E, β-carotene, zinc, iron, copper, manganese and selenium. Other authors used dietary antioxidant indicators, taking into account only some antioxidant vitamins and minerals [57,58]. In this study, higher adherence to DAQI had a significant impact on the lower impact of rosacea on QoL. The highest tertiles of DAQI, after being adjusted for confounding variables, diminished the odds about 40–45% of the moderate, large and extremely large effect of rosacea on QoL after the cosmetic procedure compared with the lowest DAQI. Moreover, the highest tertiles of DAQI diminished the odds about 7–11% of the occurrence of rosacea symptoms (pustules and papules, flushing, persistent erythema, and telangiectasia) after cosmetic procedure compared with the lowest tertiles. This study showed that dietary antioxidants can support antioxidant cosmetic treatments in improving the QoL of rosacea patients.

The present study also has some strengths and limitations. The strength of this study is that we used a standardized questionnaire (DLQI) to assess the QoL of patients and the studied group was large for an interventional study. Moreover, in this study, we developed and used a new dietary antioxidant quality index (DAQI). Additionally, there is little scientific research on the impact of cosmetic procedures and dietary antioxidants on improving the QoL of rosacea patients. The main limitation of this study is that the study group was not homogeneous in terms of gender and age. In summary, the direction of research is promising, and further research is needed to identify factors that improve the QoL of patients with rosacea.

## 5. Conclusions

This study showed that rosacea has a negative impact on QoL, but a comprehensive approach to treatment, including antioxidant cosmetic treatment and a healthy lifestyle, especially dietary antioxidants, can improve the QoL of patients with rosacea.

## Figures and Tables

**Table 1:** Baseline characteristics of the study population.

Variables	Population		
	Women N = 123	Men N = 37	Total N = 160
Age (year), X ± SD	46.3 ± 6.7	46.6 ± 5.4	46.4 ± 6.4
(range)	(32–72)	(32–72)	(32–72)
Age (year), N (%)			
32–50	78 (63.4)	23 (62.2)	101 (63.1)
51–72	45 (36.6)	14 (37.8)	59 (36.9)
Level of education, N (%)			
Higher	57 (46.3)	12 (32.4)	69 (43.1)
Middle	42 (34.2)	16 (43.3)	58 (36.3)
Bellow middle	24 (19.5)	9 (24.3)	33 (20.6)
BMI (kg/m^2^), X ± SD	24.4 ± 4.6	24.8 ± 3.6	24.5 ± 4.4
(range)	(16.1–37.5)	(19.0–36.9)	(16.1–37.5)
BMI (kg/m^2^), N (%)			
<18.5	6 (4.9)	0 (0.0)	6 (3.7)
18.5–24.9	76 (61.8)	18 (48.7)	94 (58.8)
25.0–29.9	26 (21.1)	12 (32.4)	38 (23.8)
>30.0	15 (12.2)	7 (18.9)	22 (13.7)
Current smoking, N (%)			
every day	70 (56.9)	25 (67.6)	95 (59.4)
occasionally	0 (0.0)	0 (0.0)	0
never	53 (43.1)	12 (32.4)	65 (40.6)
Alcohol drinking, N (%)			
5–7 times a week	0 (0.0)	0 (0.0)	0 (0.0)
2–4 times a week	2 (1.6)	6 (16.2)	8 (5.0)
once a week	19 (15.4)	9 (24.3)	28 (17.5)
occasionally	78 (63.4)	18 (48.7)	96 (60.0)
never	24 (19.5)	4 (10.8)	28 (17.5)
Night sleep duration (hours), N (%)			
7–9	46 (37.4)	14 (37.8)	60 (37.5)
<7 or >9	77 (62.6)	23 (62.2)	100 (62.5)
Level of physical activity, N (%)			
High	10 (8.1)	6 (16.3)	16 (10.0)
Moderate	60 (48.8)	17 (45.9)	77 (48.1)
Low	53 (43.1)	14 (37.8)	67 (41.9)
Phototype, N (%)			
I	49 (39.8)	12 (32.4)	61 (38.1)
II	60 (48.8)	22 (59.5)	82 (51.3)
III	12 (9.8)	2 (5.4)	14 (8.8)
IV	2 (1.6)	1 (2.7)	3 (1.8)
Occurrence of diseases, N (%)			
digestive system diseases	50 (40.7)	20 (54.1)	70 (43.8)
*Helicobacter pylori*	24 (19.5)	13 (35.1)	37 (23.1)
demodicosis	6 (4.9)	3 (8.1)	9 (5.6)
hormonal disorders	38 (30.9)	9 (24.3)	47 (29.4)
Duration of rosacea, N (%)			
<4 years	47 (38.2)	15 (40.2)	62 (38.8)
≥4 years	76 (61.8)	22 (59.5)	98 (61.2)
Age at onset (years), X ± SD	41.1 ± 4.5	43.8 ± 3.2	42.3 ± 4.2
(range)	(16–52)	(34–50)	(16–52)
Current skin condition, N (%)			
pustules and papules	65 (52.8)	16 (43.2)	81 (50.6)
flushing	115 (93.5)	30 (81.1)	145 (90.6)
persistent erythema	112 (91.1)	29 (78.4)	141 (88.1)
telangiectasia	119 (96.7)	32 (86.5)	151 (94.4)
phymatous changes	0 (0.0)	8 (21.6)	8 (5.0)
plaques	13 (10.6)	5 (13.5)	18 (11.3)
edema	17 (13.8)	6 (16.2)	23 (14.4)
Factors intensifying changes, N (%)			
sun exposure	93 (75.6)	21 (56.8)	114 (71.3)
temperature changes	76 (62.8)	23 (62.2)	99 (61.9)
cosmetics	103 (83.7)	32 (86.5)	135 (84.4)
alcohol	68 (55.3)	26 (70.3)	94 (58.8)
spicy foods	79 (64.2)	28 (75.7)	107 (66.9)
hot foods and drinks	57 (46.3)	25 (67.6)	82 (51.3)
stress	80 (65.0)	30 (81.1)	110 (68.8)
intense physical activity	43 (35.0)	12 (32.4)	55 (34.4)
Family history of rosacea, N (%)	81 (65.9)	28 (75.7)	109 (68.1)

Data were presented as mean ± standard deviation (X ± SD) or number and percentage (N, %), BMI—body mass index.

**Table 2:** Calculation of DAQI (dietary antioxidant quality index).

Variables	Population		
	Women N = 123	Men N = 37	Total N = 160
Energy of diet (kcal/d.), X ± SD	1572.6 ± 649.0	1723.0 ± 551.7	1602.9 ± 632.0
(range)	(580.0–4199.3)	(702.0–3240.1)	(580.0–4199.3)
DTAC (mmol/d.), X ± SD	9.3 ± 8.5	13.5 ± 9.2	10.5 ± 7.4
(range)	(3.4–13.6)	(6.4–16.3)	(3.4–16.3)
DTAC/1000 kcal, X ± SD	6.2 ± 5.2	7.4 ± 6.3	6.6 ± 5.8
≥Me, N (%)	38 (30.9)	15 (40.5)	53 (33.1)
DP (mg/d.), X ± SD	1743.2 ± 656.3	1832.4 ± 623.6	1798.2 ± 686.1
(range)	(325.1–3820.2)	(395.4–3761.8)	(325.1–3820.2)
DP/1000 kcal, X ± SD	1162.2 ± 453.3	1077.6 ± 512.5	1123.2 ± 487.6
≥Me, N (%)	42 (34.1)	18 (48.6)	60 (37.5)
DPH (mg/d.), X ± SD	245 ± 193	297 ± 243	275 ± 236
(range)	(4.5–487.3)	(7.9–543.3)	(4.5–543.3)
DPH/1000 kcal, X ± SD	163.3 ± 134.2	165.7 ± 142.8	171.8 ± 145.5
≥Me, N (%)	35 (28.5)	12 (32.4)	47 (29.4)
DL (µg/d.), X ± SD	768.7 ± 567.7	845.1 ± 786.4	812.5 ± 634.8
(range)	(300–1800)	(400–2100)	(300–2100)
DL/1000 kcal, X ± SD	512.4 ± 437.8	497.5 ± 457.4	507.5 ± 476.4
≥Me, N (%)	32 (26.0)	14 (37.8)	46 (28.8)
β-carotene (µg/d.), X ± SD	2534.5 ± 1301.5	2933.7 ± 1605.4	2725.3 ± 1420.5
(range)	(124.5–4512.6)	(135.3–4664.2)	(124.5–4664.2)
β-carotene/1000 kcal, X ±SD	1689.3 ± 1154.2	1725.4 ± 1255.8	1703.1 ± 1189.5
≥Me	83 (67.5)	20 (54.1)	103 (64.4)
Vitamin C (mg/d.), X ± SD	64.0 ± 64.1	62.9 ± 48.2	63.8 ± 61.1
(range)	(1.3–166.6)	(8.0–198.0)	(1.3–198.0)
≥90% RDA	21 (17.1)	10 (27.0)	31 (19.4)
Vitamin E (mg/d.), X ± SD	6.4 ± 5.4	8.2 ± 8.8	6.8 ± 6.2
(range)	(0.6–12.0)	(1.2–19.5)	(0.6–19.5)
≥90% AI	11 (8.9)	6 (16.2)	17 (10.6)
Zinc (mg/d.), X ± SD	5.4 ± 4.2	7.4 ± 6.3	6.8 ± 5.7
(range)	(2.1–14.5)	(2.4–16.4)	(2.1–16.4)
≥90% RDA	36 (29.3)	17 (45.9)	53 (33.1)
Iron (mg/d.), X ± SD	6.4 ± 6.2	7.1 ± 6.3	6.6 ± 5.8
(range)	(3.4–25.5)	(3.6–27.2)	(3.4–27.2)
≥90% RDA	45 (36.6)	24 (64.9)	69 (43.1)
Copper (mg/d.), X ± SD	0.6 ± 0.5	0.7 ± 0.6	0.7 ± 0.7
(range)	(0.1–2.3)	(0.2–3.4)	(0.1–3.4)
≥90% RDA	51 (41.5)	20 (54.1)	71 (44.4)
Manganese (mg/d.), X ± SD	1.2 ± 1.1	1.6 ± 1.4	1.4 ± 1.3
(range)	(0.5–3.8)	(0.7–5.3)	(0.5–5.3)
≥90% AI	46 (37.4)	19 (51.4)	65 (40.6)
Selenium (µg/d.), X ± SD	37.2 ± 24.5	38.7 ± 19.2	37.9 ± 21.1
(range)	(8.5–74.8)	(11.3–73.3)	(8.5–74.8)
≥90% RDA	23 (18.7)	12 (32.4)	35 (21.9)
DAQI, X ± SD (range)	7.8 ± 3.5 (4–11)	6.7 ± 4.2 (3–9)	7.2 ± 3.9 (3–11)

Data were presented as mean ± standard deviation (X ± SD) or number and percentage (N, %), RDA—recommended dietary allowances, AI—adequate intake, DTAC—dietary total antioxidant capacity, DP—dietary polyphenols, DPH—dietary phytosterols, DL—dietary lignans, DAQI—dietary antioxidant quality index, Me—mediane.

**Table 3:** Associations between sociodemographic and lifestyle characteristics and DLQI.

Variables	N (%)	DLQI, X ± SD (Me, IQR)	*p*-Value
Gender			
women	123 (76.9)	12.2 ± 3.9 (11.4; 4.5–15.7)	<0.001
men	37 (23.1)	8.8 ± 5.1 (8.5; 3.2–13.4)	
Age (years)			<0.001
32–50	101 (63.1)	11.2 ± 4.6 (10.8; 3.1–16.5)	
51–72	59 (36.9)	8.1 ± 3.5 (7.8; 3.7–14.2)	
Level of education			0.121
Higher	69 (43.1)	9.4 ± 5.2 (9.1; 4.4–13.2)	
Middle	58 (36.3)	9.2 ± 4.7 (8.9; 3.5–14.6)	
Bellow middle	33 (20.6)	8.8 ± 4.5 (8.4; 4.1–13.1)	
BMI (kg/m^2^)			<0.001
18.5–24.9	94 (58.8)	8.4 ± 4.3 (7.9; 4.5–13.4)	
<18.5 or >24.9	66 (41.2)	10.5 ± 4.4 (9.5; 4.9–15.2)	
Current smoking			<0.001
Yes	95 (59.4)	11.3 ± 4.9 (10.7; 3.9–14.6)	
No	65 (40.6)	8.6 ± 4.7 (8.4; 3.5–12.5)	
Alcohol drinking			
2–4 times a week	8 (5.0)	10.9 ± 4.5 (10.4; 4.2–15.8)	<0.001
once a week	28 (17.5)	10.2 ± 4.3 (9.9; 4.1–14.8)	
occasionally	96 (60.0)	8.4 ± 5.5 (8.2; 4.3–14.2)	
never	28 (17.5)	8.1 ± 5.3 (7.7; 3.4–12.9)	
Night sleep duration (h)			
7–9	60 (37.5)	8.2 ± 4.7 (7.9; 3.4–13.8)	<0.001
<7 or >9	100 (62.5)	11.3 ± 5.3 (10.8; 3.8–14.6)	
Level of physical activity			0.545
moderate	77 (48.1)	9.5 ± 4.8 (9.3; 4.6–14.5)	
low or high	83 (51.9)	9.3 ± 4.5 (8.9; 4.7–15.1)	
DAQI			
1–4 points	65 (40.6)	12.6 ± 5.8 (11.3; 3.5–15.4)	<0.001
5–8 points	52 (32.5)	8.7 ± 4.9 (8.3; 3.4–13.9)	
9–12 points	43 (26.9)	7.2 ± 4.6 (7.6; 3.7–11.5)	

Data were presented as mean ± standard deviation (X ± SD) or number and percentage (N, %), Me—median, IQR—interquartile range, DLQI—dermatology life quality index, DAQI—dietary antioxidant quality index, BMI—body mass index.

**Table 4:** Associations between sociodemographic and lifestyle characteristics according to DAQI tertiles.

Variables	DAQI			*p*-Value
	Low (1–4), N = 65	Moderate (5–8), N = 52	High (9–12), N = 43	
Sex				<0.001
Women	39 (60.0)	41 (78.8)	43 (100)	
Men	26 (40.0)	11 (21.2)	0 (0.0)	
Age (years)	47.5 ± 6.9	46.1 ± 6.6	45.2 ± 6.3	<0.001
Level of education				0.245
Higher	21 (32.3)	28 (53.8)	20 (46.5)	
Middle	20 (30.8)	21 (40.4)	17 (39.5)	
Bellow middle	24 (36.9)	3 (5.8)	6 (14.0)	
BMI (kg/m^2^)	25.3 ± 4.6	24.1 ± 4.4	23.2 ± 4.8	<0.001
Current smoking				<0.001
Yes	46 (70.8)	30 (57.7)	19 (44.2)	
No	19 (29.2)	22 (42.3)	24 (55.8)	
Alcohol drinking				
5–7 times a week	0 (0.0)	0 (0.0)	0 (0.0)	0.074
2–4 times a week	5 (7.7)	2 (3.8)	1 (2.3)	
once a week	5 (7.7)	16 (30.8)	7 (16.3)	
occasionally	47 (72.3)	25 (48.1)	24 (55.8)	
never	8 (12.3)	9 (17.3)	11 (25.6)	
Night sleep duration (h)				0.545
7–9	14 (21.5)	29 (55.8)	17 (39.5)	
<7 or >9	51 (78.5)	23 (44.2)	26 (60.5)	
Physical activity				0.356
moderate	39 (60.0)	15 (28.8)	23 (53.5)	
low or high	26 (40.0)	37 (71.2)	20 (46.5)	

Data were presented as mean ± standard deviation (X ± SD) or number and percentage (N, %), BMI—body mass index, DAQI—dietary antioxidant quality index.

**Table 5:** Multivariate adjusted odds ratios (ORs) and 95% confidence intervals (CIs) for the impact of rosacea on QoL after cosmetic procedure across tertiles of DAQI.

Impact of Rosacea on QoL	DAQI		
	Low (1–4) N = 65	Moderate (5–8) N = 52	High (9–12) N = 43
DLQI	8.7 ± 5.3	7.2 ± 4.6	6.4 ± 3.8
	*p* < 0.001		
DLQI < 6 points			
Crude OR (95% CI) ^1^	1	1.23 (0.45–3.32)	0.78 (0.34–2.11)
Adjusted OR (95% CI) ^2^	1	1.54 (0.48–4.65)	0.81 (0.35–2.56)
Adjusted OR (95% CI) ^3^	1	1.48 (0.49–4.11)	0.86 (0.33–2.87)
DLQI = 6–10 points			
Crude OR (95% CI) ^1^	1	0.51 (0.20–0.85) *	0.54 (0.23–0.88) *
Adjusted OR (95% CI) ^2^	1	0.52 (0.22–0.94) *	0.53 (0.22–0.91) *
Adjusted OR (95% CI) ^3^	1	0.54 (0.21–0.97) *	0.55 (0.23–0.92) *
DLQI > 10 points			
Crude OR (95% CI) ^1^	1	0.47 (0.25–0.91) *	0.54 (0.24–0.89) *
Adjusted OR (95% CI) ^2^	1	0.46 (0.25–0.94) *	0.57 (0.21–0.92) *
Adjusted OR (95% CI) ^3^	1	0.47 (0.24–0.96) *	0.60 (0.22–0.97) *

DAQI—dietary antioxidant quality index, DLQI—The Dermatology Life Quality Index, ^1^—Model 1—crude data, ^2^—Model 2—adjusted for age, sex, daily energy intake, ^3^—Model 3—adjusted for age, sex, daily energy intake, BMI, smoking, *—*p* < 0.05.

**Table 6:** Multivariate adjusted odds ratios (ORs) and 95% confidence intervals (CIs) for the occurrence of rosacea symptoms after cosmetic procedure across tertiles of DAQI.

Occurrence of Rosacea Symptoms after ACP	DAQI		
	Low (1–4) N = 65	Moderate (5–8) N = 52	High (9–12) N = 43
Pustules and papules, N (%)	29 (44.6)	20 (38.5)	14 (32.6)
	*p* < 0.008		
Crude OR (95% CI) ^1^	1	0.98 (0.92–1.29)	0.89 (0.73–0.97) *
Adjusted OR (95% CI) ^2^	1	0.93 (0.89–1.12)	0.91 (0.76–0.97) *
Adjusted OR (95% CI) ^3^	1	0.89 (0.83–1.11)	0.92 (0.75–0.98) *
Flushing, N (%)	38 (58.5)	27 (51.9)	18 (41.9)
	*p* < 0.019		
Crude OR (95% CI) ^1^	1	0.87 (0.78–0.98) *	0.91 (0.74–0.94) *
Adjusted OR (95% CI) ^2^	1	0.84 (0.81–1.08)	0.88 (0.71–0.95) *
Adjusted OR (95% CI) ^3^	1	0.86 (0.84–1.15)	0.93 (0.72–0.97) *
Persistent erythema, N (%)	42 (64.6)	31 (59.6)	22 (51.2)
	*p* < 0.011		
Crude OR (95% CI) ^1^	1	0.87 (0.75–0.93) *	0.85 (0.74–0.93) *
Adjusted OR (95% CI) ^2^	1	0.76 (0.74–1.13)	0.86 (0.71–0.95) *
Adjusted OR (95% CI) ^3^	1	0.77 (0.72–1.15)	0.89 (0.75–0.96) *
Teleangiectasia, N (%)	44 (67.7)	32 (61.5)	24 (55.8)
	*p* < 0.014		
Crude OR (95% CI) ^1^	1	0.78 (0.69–0.91) *	0.88 (0.74–0.98) *
Adjusted OR (95% CI) ^2^	1	0.76 (0.74–1.14)	0.92 (0.71–0.96) *
Adjusted OR (95% CI) ^3^	1	0.77 (0.74–1.26)	0.91 (0.72–0.97) *

DAQI—dietary antioxidant quality index, ACP—antioxidant cosmetic procedure, ^1^—Model 1.—crude data, ^2^—Model 2—adjusted for age, sex, daily energy intake, ^3^—Model 3—adjusted for age, sex, daily energy intake, BMI, smoking, *—*p* < 0.05.

## Data Availability

The datasets are not publicly available because the individual privacy of the participants should be protected. Data are available from the corresponding author upon reasonable request.
